# Supplementary material for: Fecal glucocorticoid metabolite and T3 profiles of orphaned elephants differ from non-orphaned elephants in Zambia
Source: PeerJ. 2025 Apr 4;13:e19122. doi: 10.7717/peerj.19122 (PMC11974515; doi:10.7717/peerj.19122)
Supplement: Supplemental Information 3 [file peerj-13-19122-s003.docx]

**Supplemental File**

**Table S1.** Demographics of the study population. * Denotes an orphan that was already released back into the wild at the time of sample collection. The remaining orphaned elephants were still under human care at the time of sample collection. ED: early-dry season; LD: late-dry season; Rea Orph: Reason for orphaning; HEC: Human elephant conflict; Unk.: Unknown; Poac.: Poaching; CS: Dung certainty scores are defined as follows: 0=Dung that was randomly found with no elephants necessarily around; 1=Dung collected from a specific herd, but the individual was unknown; 2= Dung collected from a specific spot an individual was observed occupying but the actual defecation was not observed; and 3=Dung collected from an observed individual defecating. All orphan samples had a certainty score of 3, and their age corresponds to their first sample collection date.

| Orphaned Elephants | | | | | | Control Elephants during early-dry season | | | | | Control Elephants during late-dry season | | | | |
| --- | --- | --- | --- | --- | --- | --- | --- | --- | --- | --- | --- | --- | --- | --- | --- |
| ID | Age (mo.) | Age  Class | Sex | Season Collected | Rea  Orph | ID | Age (mo.) |  | Sex | CS | ID | Age (mo.) |  | Sex | CS |
| Batoka* | 157 | SA | M | ED | Unk | 5 | 84 | JUV | Unk. | 1 | 85 | 24 | INF | Unk. | 1 |
| Chamilandu | 181 | A | F | ED; LD | Poac | 6 | 72 | JUV | Unk. | 1 | 86 | 96 | SA | Unk. | 1 |
| Chipembele | 26 | INF | M | LD | HEC | 8 | 180 | SA | Unk. | 1 | 87 | 60 | JUV | F | 3 |
| Kasewe | 68 | JUV | F | ED; LD | Unk | 9 | 96 | SA | Unk. | 1 | 88 | 132 | SA | F | 3 |
| Kavalamanja | 127 | SA | F | ED; LD | Poac | 11 | 108 | SA | M | 3 | 89 | 144 | SA | M | 3 |
| Fungulani | 42 | INF | F | ED; LD | Unk | 12 | 36 | INF | Unk. | 1 | 90 | 120 | SA | Unk. | 1 |
| Ludaka | 48 | JUV | M | ED; LD | Unk | 13 | 132 | SA | Unk. | 1 | 91 | 120 | SA | Unk. | 1 |
| Maramba | 133 | SA | M | ED; LD | Unk | 14 | 96 | SA | Unk. | 1 | 95 | 72 | JUV | Unk. | 3 |
| Mbila | 15 | INF | F | ED; LD | HEC | 15 | 4 | INF | F | 1 | 96 | 48 | JUV | Unk. | 1 |
| Mkaliva | 60 | JUV | F | ED; LD | Poac | 16 | 24 | INF | Unk. | 1 | 101 | 18 | INF | Unk. | 1 |
| Mosi | 129 | SA | M | ED; LD | Unk | 17 | 144 | SA | Unk. | 1 | 105 | 72 | JUV | F | 3 |
| Mphamvu | 98 | SA | M | ED; LD | Unk | 18 | 84 | JUV | Unk. | 1 | 107 | 168 | SA | M | 3 |
| Muchichili | 82 | JUV | M | ED; LD | Unk | 23 | 120 | SA | Unk. | 3 | 108 | 108 | SA | Unk. | 3 |
| Mulisani | 74 | JUV | M | ED; LD | Poac | 24 | 60 | JUV | Unk. | 1 | 111 | 168 | SA | Unk. | 3 |
| Musolole | 120 | SA | M | ED; LD | Poac | 26 | 96 | SA | Unk. | 3 | 112 | 168 | SA | Unk. | 3 |
| Nkala | 98 | SA | M | ED; LD | HEC | 32 | 168 | SA | F | 1 | 113 | 120 | SA | Unk. | 3 |
| Olimba | 30 | INF | F | ED; LD | Poac | 33 | 132 | SA | F | 3 | 115 | 168 | SA | Unk. | 3 |
| Rufunsa | 131 | SA | M | ED; LD | Poac | 34 | 132 | SA | M | 3 | 116 | 120 | SA | Unk. | 3 |
| Tafika* | 150 | SA | M | ED; LD | HEC | 40 | 5 | INF | Unk. | 1 | 117 | 84 | SA | Unk. | 3 |
| Lufutuko | 42 | INF | F | ED; LD | HEC | 41 | 132 | SA | Unk. | 1 | 118 | 60 | SA | Unk. | 1 |
| Shezongo | 14 | INF | M | ED; LD | HEC | 42 | 96 | SA | Unk. | 1 | 123 | 72 | JUV | M | 3 |
|  |  |  |  |  |  | 43 | 120 | SA | Unk. | 1 | 124 | 84 | SA | Unk. | 3 |
|  |  |  |  |  |  | 44 | 108 | SA | Unk. | 1 |  |  |  |  |  |
|  |  |  |  |  |  | 45 | 24 | INF | Unk. | 1 |  |  |  |  |  |
|  |  |  |  |  |  | 46 | 60 | JUV | Unk. | 1 |  |  |  |  |  |
|  |  |  |  |  |  | 48 | 96 | SA | Unk. | 1 |  |  |  |  |  |
|  |  |  |  |  |  | 49 | 132 | SA | F | 3 |  |  |  |  |  |
|  |  |  |  |  |  | 50 | 84 | JUV | M | 3 |  |  |  |  |  |
|  |  |  |  |  |  | 51 | 120 | SA | M | 3 |  |  |  |  |  |
|  |  |  |  |  |  | 52 | 72 | JUV | Unk. | 1 |  |  |  |  |  |
|  |  |  |  |  |  | 53 | 8 | INF | F | 2 |  |  |  |  |  |
|  |  |  |  |  |  | 54 | 84 | JUV | Unk. | 1 |  |  |  |  |  |
|  |  |  |  |  |  | 55 | 72 | JUV | Unk. | 1 |  |  |  |  |  |
|  |  |  |  |  |  | 57 | 120 | SA | Unk. | 1 |  |  |  |  |  |
|  |  |  |  |  |  | 58 | 72 | JUV | Unk. | 1 |  |  |  |  |  |
|  |  |  |  |  |  | 59 | 72 | JUV | Unk. | 1 |  |  |  |  |  |
|  |  |  |  |  |  | 60 | 24 | INF | F | 3 |  |  |  |  |  |
|  |  |  |  |  |  | 61 | 18 | INF | Unk. | 1 |  |  |  |  |  |
|  |  |  |  |  |  | 62 | 96 | SA | Unk. | 1 |  |  |  |  |  |
|  |  |  |  |  |  | 63 | Unk. | - | Unk. | 1 |  |  |  |  |  |
|  |  |  |  |  |  | 64 | 36 | INF | F | 3 |  |  |  |  |  |
|  |  |  |  |  |  | 65 | 168 | SA | F | 3 |  |  |  |  |  |
|  |  |  |  |  |  | 67 | 184 | A | Unk. | 1 |  |  |  |  |  |
|  |  |  |  |  |  | 68 | 144 | SA | Unk. | 1 |  |  |  |  |  |
|  |  |  |  |  |  | 69 | 132 | SA | M | 1 |  |  |  |  |  |
|  |  |  |  |  |  | 70 | 96 | SA | Unk. | 1 |  |  |  |  |  |
|  |  |  |  |  |  | 71 | 108 | SA | Unk. | 1 |  |  |  |  |  |
|  |  |  |  |  |  | 72 | 84 | JUV | Unk. | 1 |  |  |  |  |  |
|  |  |  |  |  |  | 73 | 108 | SA | M | 3 |  |  |  |  |  |
|  |  |  |  |  |  | 74 | 84 | JUV | Unk. | 1 |  |  |  |  |  |
|  |  |  |  |  |  | 75 | 84 | JUV | Unk. | 1 |  |  |  |  |  |
|  |  |  |  |  |  | 76 | 72 | JUV | M | 3 |  |  |  |  |  |
|  |  |  |  |  |  | 77 | 8 | INF | M | 1 |  |  |  |  |  |
|  |  |  |  |  |  | 79 | 144 | SA | M | 3 |  |  |  |  |  |
|  |  |  |  |  |  | 80 | 84 | JUV | Unk. | 1 |  |  |  |  |  |
|  |  |  |  |  |  | 81 | 60 | JUV | Unk. | 1 |  |  |  |  |  |
|  |  |  |  |  |  | 82 | 60 | JUV | Unk. | 1 |  |  |  |  |  |
|  |  |  |  |  |  | 83 | 120 | SA | Unk. | 1 |  |  |  |  |  |

**Table S2.** Dietary details for orphaned elephants at GRI’s Lilayi Elephant Nursery (LEN) and Kafue Release Facility (KRF). Underlined time points correspond to underlined ages at LEN. LEN houses elephants from time of rescue until 3-4 years of age. KRF houses elephants from 3-4 years of age until their soft release into Kafue National Park.

|  |  | **LEN** | | | **KRF** | | |  |
| --- | --- | --- | --- | --- | --- | --- | --- | --- |
| **Time of feeding** | **Feed** | **1-3 mo of age** | **3 mo- 1 yr of age** | **1-3 yrs of age** | **3-4 yrs of age** | **4-6 yrs of age** | **>6 yrs of age** | **Feed contents** |
| 6:00am, 9:00am, 12:00pm, 2:00/3:00pm, 5:00/6:00pm, 9:00pm, 12:00am, 3:00am | Milk bottle | Yes | Yes | Yes | Yes | No | No | Coconut powder, coconut oil, poultry amino acids, human infant formula, oats, moringa, water |
| 6:00am, 12:00pm, 2:00pm, 5:00pm, 12:00am | Power Ball/ Milk | No | No | No | No | Yes | No |  |
| 6:00am, 2:00pm, 5:00pm | Power Ball | No | No | No | No | Yes | Yes | Same contents as milk bottle, but without water |
| 12:00pm, 5:00pm | Pellets | No | No | No | Yes | Yes | Yes | Pellets produced for pigs (Nutrifeed) |
| 12:00pm, 5:00/6:00pm | Browse | Yes | Yes | Yes | Yes | Yes | Yes | Various miombo woodland cut branches from the local environment (e.g., *Brachstegia utilis,Brachstegia boehmii,Combretum molle,Diplorhynchus condylocarpon*) |


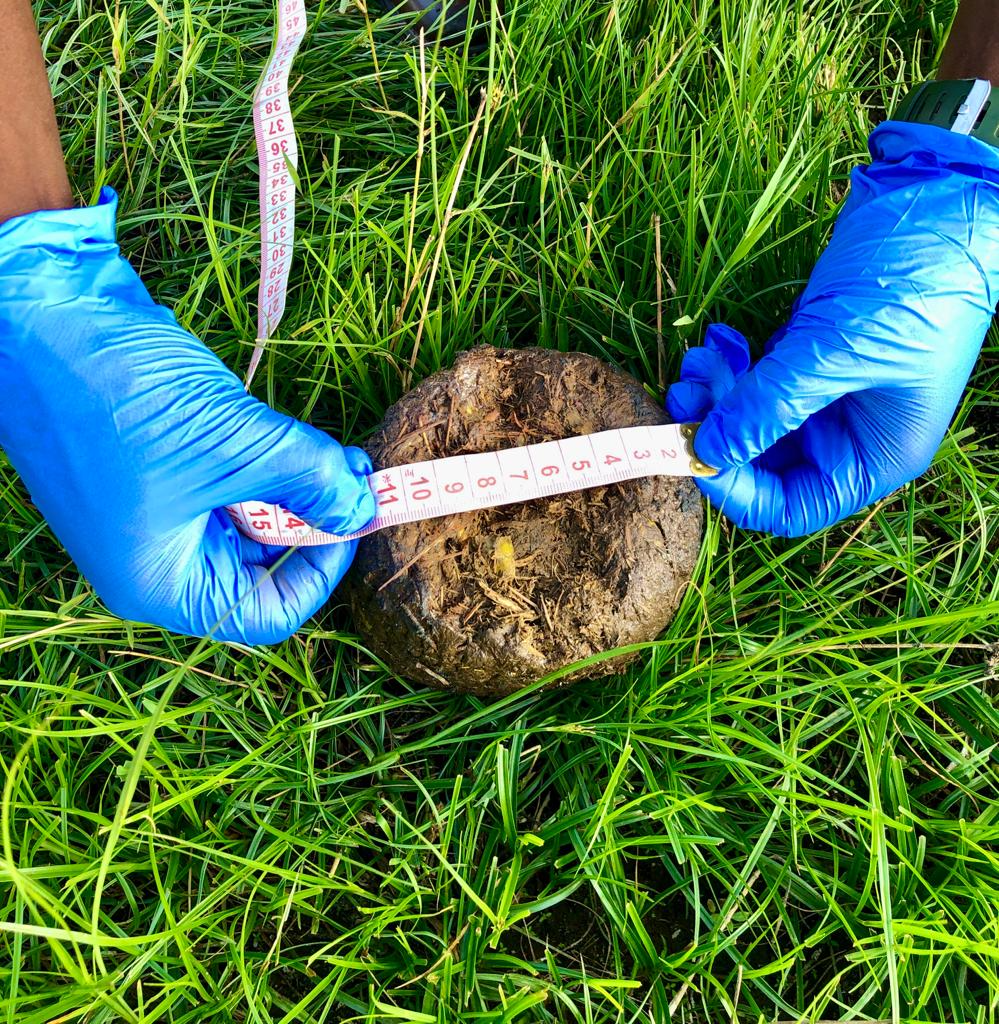


**Figure S1.** Photo with schematic insert of how dung diameter was measured. The diameter, represented by the black solid and dotted lines, on both sides (A and B) of the dung ball were measured on two intact dung balls, and then averaged to estimate the elephants’ age.
